# Life History Plasticity of a Tropical Seabird in Response to El Niño Anomalies during Early Life

**DOI:** 10.1371/journal.pone.0072665

**Published:** 2013-09-04

**Authors:** Sergio Ancona, Hugh Drummond

**Affiliations:** Departamento de Ecología Evolutiva, Instituto de Ecología, Universidad Nacional Autónoma de México, México DF, México; CNRS, Université de Bourgogne, France

## Abstract

Food shortage and other challenges associated with El Niño Southern Oscillation (ENSO) experienced early in life may have long-term impacts on life history traits, but these potential impacts remain virtually unexplored. By monitoring 2556 blue-footed boobies from 11 cohorts, we showed that birds facing warm water ENSO conditions (and probably low food availability) in the natal year were underweight at fledging, recruited earlier and bred less frequently, but showed no deficit in longevity or breeding success over the first 10 years. Life history impacts of ENSO were substantial when experienced in the prenatal year, the natal year, or the second year of life, and absent when experienced in the third year of life, implying that harsh conditions have greater effects when experienced earlier in life. Sexual differences in impacts depended on the age when warm water conditions were experienced: pre-natal and natal experience, respectively, induced early recruitment and influenced the relationship between age and laying date only in females, whereas second year experience reduced total breeding success only of males. Most surprising were positive transgenerational impacts in females: daughters of females that experienced ENSO conditions in their natal year showed improved breeding success. Developmental plasticity of boobies thus enables them to largely neutralize potential long-term impacts of harsh climatic conditions experienced early in life.

## Introduction

Nutritional deficits and other environmental challenges (e.g., population density, parasite loads, weather) experienced during prenatal or postnatal development shape physiological and structural traits, affecting body size at independence and adult characteristics [1]–[3]. Although affected individuals may catch up and achieve normal size when conditions improve, they can pay delayed costs of initial adversities or compensatory growth that follows ([2], [4]). Costs can be expressed early in terms of higher mortality after birth or lower probability of recruitment [5]–[7], but can also persist into old age ([8]–[10] but see [11]), for example as delayed breeding [12], reduced longevity or lifetime reproductive success [13]–[14] or a higher rate of senescence [12], [15]. Thus, early stresses can affect life-history traits by influencing breeder quality [16] and costs of reproduction [17], [18].

The earlier an environmental perturbation takes place during development the greater its potential effects [1], and perturbations experienced early in life often affect the sexes differently [19]. Sex-specific effects of rearing conditions can be due to sexual differences in nutritional requirements and sensitivity to conditions during development [20]–[22]. Moreover, selection may act differently on each sex [10], [23], leading to different sex-specific optima for trait values and trade-offs [24]. Importantly, the magnitude of sexual differences can be associated with the intensity of sexual selection [13]. Challenges can be experienced through parental effects: harsh conditions experienced by a parent (particularly the mother) at any stage of development including adulthood can affect phenotypes of the next generation (transgenerational effects [25], [26]). For instance, adult daughters of female zebra finches reared in experimentally enlarged broods are undersized and show diminished reproductive success [27]. Similarly, quality of the natal territory and duration of the rearing period can affect the number or quality of future offspring in wild bird populations [9], [28]. Such transgenerational effects of rearing conditions have been documented in several species [1], [29], including humans [30], and may be transmitted by epigenetic mechanisms [31] likely mediated by endocrine systems [26]. Transgenerational effects may also arise if harsh developmental conditions constrain rearing capabilities of affected individuals [32].

Costs of exposure to early stress can trade off against each other [33]–[34] and developmental programmes often prioritize compensatory growth or reproduction over longevity [14], apparently because selection favours benefits experienced early in life when selection is stronger [2], [35]. For instance, captive female guppies compensate for experimental food restrictions during the juvenile stage by accelerating growth rates in the adult stage and are able to devote more resources to reproduction than unrestricted females [36]. Similarly, wild-caught and domestic zebra finches raised in experimentally enlarged broods mature faster and do not exhibit deficits in breeding success despite being undersized compared to birds raised in diminished broods [37]. However, these compensatory responses of guppies and zebra finches may compromise their survival and longevity [36], [38].

In wild populations, negative impacts of harsh natal environments on offspring may sometimes be avoided if development is buffered against natural challenges [11], for example through flexible parental care [39] or through learning and social integration of juveniles during the pre-breeding stage [40], [41]; or if life-history trajectory is optimized through developmental plasticity [10]. Such developmental buffering could mitigate the impacts of a poor start in life on subsequent reproduction, but its benefits are expected to be evident in early-life breeding attempts and disappear later on [3]. However, negative impacts on subsequent reproduction may be masked whenever harsh environments result in high quality individuals [42] being over-represented among successful breeders, if their offspring are also of high quality [43].

Among the natural climatic fluctuations that numerous species are exposed to during different stages of their lives are the North Atlantic Oscillation (NAO) and El Niño Southern Oscillation (ENSO). These patterns of climate variability mediate variation in regional and local parameters of land and ocean temperature, winds, ocean currents and precipitation, affecting growth, survival and fecundity in animal populations [44] by directly influencing organisms' physiology and metabolism [45], or by inducing changes in food availability [46]–[48]. While numerous studies have documented the short-term impacts of NAO and ENSO that occur in the same season or after a lag of a few months or years (reviewed in [44]), the long-term fitness consequences arising from effects on prenatal or postnatal development have seldom been examined. Potentially, life-history trajectories and population dynamics could be affected [49], leading to variation among cohorts in fitness components [44]. Furthermore, impacts of these drivers of global climate on early development could have cascading transgenerational effects that have not hitherto been explored.

Evidence of long-term fitness consequences of climatic conditions experienced early in life comes from long-term observations of ungulates and seabirds in temperate regions. Red deer (*Cervus elaphus*) that experience severe winter conditions driven by NAO before or shortly after birth develop into undersized adults [50] with diminished lifetime reproductive success [51]. Similarly, Soay sheep (*Ovies aries*) that encounter severe winter conditions associated with the NAO during their prenatal and postnatal development are lighter at birth and show delayed sexual maturation [52]. NAO-related variations in land and sea temperatures affected recruitment rates of northern fulmars (*Fulmarus glacialis*) [53], and ENSO anomalies prejudiced survival of immature black-browed albatrosses (*Thalassarche melanophrys*) without affecting the timing of recruitment, possibly because the long maturation period of these birds (5–15 years) weakened the importance of natal conditions [54]. But we lack a general appreciation of the long-term fitness consequences of ENSO conditions experienced early in life and, more generally, of long-term effects in tropical populations. In addition, it is not known whether long-term effects of ENSO differ between the sexes and how they vary with the developmental stage when exposure occurs (e.g., embryo, neonate juvenile or pre-breeder).

In this study, we tested for long-term developmental effects of ENSO on the blue-footed booby, a long-lived, locally foraging seabird that breeds in topical waters [55] and whose nestling growth (i.e., body mass at age 34 days, roughly half way through growth), recruitment, adult survivorship and reproduction decrease when the warm waters associated with ENSO deplete ocean productivity and prey availability [56], [57]. Using longitudinal data on 11 cohorts up to age 10 years, we investigated whether ENSO conditions during prenatal or early postnatal development have persistent effects on life-history traits after recruitment and whether the effects documented in females cascade to the next generation, influencing life-history traits of their recruited daughters and sons. We expected female and male boobies to recruit at older ages, establish their clutches later in the season, breed fewer times and show diminished reproductive success or die younger when warm water conditions prevailed in the year before their birth (potentially affecting their parents before laying [25]) or in any of the first three years of life before recruitment. We also expected oceanographic conditions in the year before birth or the natal year (prenatal and early postnatal conditions) to have greater effect than conditions in the second or third year of life.

Additionally, we asked whether natal oceanographic conditions affect size and body mass of recruits at fledging and whether these growth differences impact fitness of recruits. If nestling growth of recruits varies with ocean temperatures in their natal years, then correlations between temperature and life history traits are more likely due to developmental plasticity than to differential breeding of high quality individuals in poor years.

## Materials and Methods

### Ethics statement

All fieldwork conducted complied with animal welfare regulations in Mexico and was annually supervised and approved by Dirección General de Vida Silvestre, Secretaría de Gestión para la Protección Ambiental (SEMANART approval reference numbers 517, 574, 5664, 10470, SGPA/DGVS/01323, SGPA/DGVS/3152, SGPA/DGVS/1543, SGPA/DGVS/0491, SGPA/DGVS/1547, SGPA/DGVS/10832, SGPA/DGVS/01916, SGPA/DGVS/00733, SGPA/DGVS/00357, SGPA/DGVS/00505, SGPA/DGVS/00091).

### Study species

We studied the colony of blue-footed boobies on Isla Isabel, an island off the Pacific coast of Mexico, at the southern boundary of the Gulf of California (21°52′N, 105°54′W). These socially monogamous seabirds breed colonially on islands in the eastern tropical Pacific [55]. They plunge-dive for sardines, anchovies and herrings [58] within 30 km of their breeding colonies [59], capturing fewer types of prey when warm ENSO conditions prevail during the breeding season [58]. They lay 1–3 eggs and fledge 0–3 chicks after 41–49 days incubation and three to four months of biparental feeding [60]. Females, which grow faster and become larger and 32 per cent heavier than males when adults [61], provide a greater mass of food to chicks, whereas males assume most of the costs of establishing and defending a nesting territory [60].

On Isla Isabel, the progeny sex ratio is male-biased (51–56%) and is similar at hatching, fledging and recruitment [56], [62], [63]. The two sexes show long-term fidelity to their first breeding sites (close to their natal sites), and rarely disperse long distances from their natal colony [64], making it possible to obtain complete records of individual life histories. Most females and males recruit at age 2–6 years, the average age for females being nearly half a year younger than for males (3.85 vs 4.32 years [11], [64]). Isla Isabel boobies can live 20 yrs or more. Both sexes produce progressively earlier clutches up to age 8–11 years; beyond that age, they lay progressively later and with declining breeding success [65]–[67], although they can recover from age-related decline by skipping breeding events [68]. Rarely, individuals breed twice in a single season. Reproduction starts between December and February, but laying and hatching of eggs continue through early June and the last fledglings reach independence at the end of July [69].

### Population monitoring and sampling

Every year from 1988 to 2010, reproduction was monitored in two study areas (20800 m^2^ and 6089 m^2^) by inspecting the contents of all nests (sites with a clutch or brood) every 3 or 6 days during the 5-month season (roughly February 20 to July 20). Laying dates of clutches were observed or estimated from hatching dates or sizes of chicks at first encounter. Chicks were considered fledglings at age 70 days old, and each one was weighed (± 20 g), measured (ulna length ± 1.0 mm) and marked with a steel ring (details in [11]). Fledglings were not sexed until they returned as breeders to the study population, when they were sexed by voice (females grunt, males whistle).

Our sample consisted of 1189 female and 1367 male recruits from 11 cohorts between 1988 and 2000 that bred for the first time at ages 3–6 years and repeatedly up to age 10 years, just before onset of reproductive senescence (data available from the Dryad Digital Repository: http://dx.doi.org/10.5061/dryad.2mg77). No fledglings were banded in 1990, and no chicks fledged in 1992, due to a severe El Niño episode. We limited the sample to birds that recruited at age 3–6 years in order to analyse the effects of oceanographic conditions experienced during each of the first 3 years of life, and because most of the few boobies that recruit after age 6 years breed only once, providing insufficient data to examine longitudinal patterns of breeding performance [67]. Some data were not available for all focal recruits, thus, some analyses must be performed with reduced sample sizes (see “RESULTS”).

Oceanographic conditions experienced by recruits in early life were indexed by sea surface temperature anomalies (SSTA,°C) in the waters surrounding Isla Isabel. SSTA are departures from the long-term seasonal mean for a particular geographical region and a fine-scale correlate of the continuous variation in the atmospheric pressure differential across the Pacific that generates ENSO [70]. Positive values of SSTA denote warm El Niño conditions in the Pacific Ocean; negative values denote cold La Niña conditions. We obtained monthly average values of SSTA at 21.5° N, 105.5° W (55 km southeast of Isla Isabel, the closest station) from the International Research Institute for Climate and Society (irithree.ldeo.columbia.edu website. Available: http://iridl.ldeo.columbia.edu/SOURCES/.NOAA/.NCEP/.EMC/.CMB/.GLOBAL/.Reyn_SmithOIv2/.monthly/. Accessed 2012 Jun 29).

### Statistical analyses

#### a) Computed variables

Recruiting age (3, 4, 5 or 6 years) was when focal individuals were recorded breeding for the first time in the study site. We computed the Julian laying date (the first-egg date) of every clutch established by focal recruits up to age 10 years. For this period, we computed for each focal recruit the total number of breeding attempts, the number of fledglings in each attempt (standardized within years by the z-transformation, using the annual population-wide mean and standard deviation from all observed breeding pairs) and the total number of fledglings. 10-year longevity of each recruit was its age at the last observed breeding record in the 10 seasons following its natal year.

We averaged the 12 monthly values of local SSTA for the calendar year before birth and for each of the first 3 calendar years of life of each focal recruit. These annual average temperature anomalies of the 11 years considered were unrelated to each other (Pearson's correlation tests; *r* values: range: −0.44–0.19; *P* values: range: 0.17–0.84) and no significant linear trends were detected in this variable during the study period (SSTA_(1988–2000)_: LM: *β* = 0.06±0.03; *F*_1,9_  = 3.19, *P* = 0.11).

We analysed females and males separately in order to avoid pseudoreplication of focal individuals that bred together during the study period and because the sexes differ in age of recruitment and in how recruiting age relates to number of breeding attempts, breeding success and longevity (e.g. [67]).

#### b) Effects on life-history traits

To test whether recruiting age, total number of breeding attempts, total breeding success (i.e., total number of fledglings) and longevity up to age 10 years are associated with mean SSTA in the year before birth or any of the first 3 years of life (fixed effects), we built linear mixed models with Poisson error distribution (GLMM). In analyses of total breeding success, however, we used zero-inflated Poisson models because excessive zero values prevented this count from satisfying the variance-mean relationship of the typical Poisson error structure [71]. In all models, we included cohort as a random effect to account for unmeasured environmental conditions in the recruits' natal years (e.g., population density or parasitism). Because cohort- and sex-specific relationships between recruiting age and number of breeding attempts, breeding success and longevity are expected in this species [67], we fitted recruiting age as an additional fixed effect in models for these traits. In addition, we included the total number of breeding attempts as a fixed effect in models for total breeding success and 10-year longevity, and examined the association between total breeding success and 10-year longevity.

#### c) Effects on laying dates

To test whether (1) laying date in the recruiting season and (2) laying dates across all breeding attempts up to age 10 years are associated with mean SSTA in the year before birth or any of the first 3 years of life, we built linear mixed models with normal error distributions (LMEs). In these LMEs, we included cohort, year of reproduction and individual identity (ring number) as random effects. In all these models, two-way interactions of interest were fitted: mean SSTA in the prenatal year and each of the first 3 years of life x current age (both linear and quadratic terms of age in LMEs with repeated measures because a quadratic effect of age on laying date has been documented in these boobies [67]) and x mean SSTA in the winter of the current season, which influences laying date in this species [57]. In models for laying dates across all breeding attempts up to age 10 years, we also fitted recruiting age as a fixed effect. We could not include identities and ages of breeding partners in the models because some birds in the study areas were not marked. The duration of pair bonds is short (on average 1.7 years) and ages of mated breeders are not correlated in the study population [66], [67]. Thus, we can assume that the degree of non-independence due to correlation of female and male identities across the duration of the pair bond and assortative mating by age was minor.

#### d) Effects on annual breeding success

We tested for associations between (1) breeding success in the recruiting season and (2) breeding success across all breeding attempts up to age 10 years (standardized within years to account for interannual variability in breeding success and produce a normal error distribution) and SSTA in the prenatal year or any of the first 3 years of life by building LMEs, with cohort and individual identity as random effects. Laying date of each breeding attempt was fitted as a fixed effect since fledging success diminishes as the season progresses on Isla Isabel [69]. Two-way interactions of interest were included in these models: mean SSTA in the prenatal year and each of the first 3 years of life x current age (both linear and quadratic terms of age in models with repeated measures since there is a quadratic effect of age on fledgling production in these boobies [65], [66]) and x the mean SSTA in winter-spring of the current season (warming of local waters in winter and spring of the current season is associated with decline in nest success in these boobies [57]). Recruiting age and two-way interactions between the linear and quadratic terms of current age x 10-year longevity were also included in models for breeding success across all breeding attempts up to age 10 years.

#### e) ENSO effects on growth of recruits

We tested for associations between SSTA experienced by focal recruits in their natal years and body mass and ulna length at fledging, using a linear model (LM) that included the interaction between SSTA and sex of recruit. After finding that natal temperature influenced body mass at fledging in both sexes, we tested whether body mass was associated with recruiting age, 10-year total number of breeding attempts, 10-year total breeding success and 10-year longevity (dependent variables) by building generalized linear models (GLMs) with Poisson error distributions. All included the interaction between body mass and sex.

#### f) Transgenerational effects

After finding that SSTA in the natal year was retained in all models analysing traits of females affected by conditions during development (i.e., models for recruiting age, number of breeding attempts and laying date; see “RESULTS”), we included mean SSTA during the natal year of each recruit's mother as an additional fixed effect to test for transgenerational maternal effects of ENSO on those same traits and also on daughters' and sons' standardized annual breeding success in the season when they recruited and across all their breeding attempts up to age 10 years, and on their total breeding success up to that age. In all these models, we included the cohort of each recruit's mother as an additional random effect in order to avoid pseudoreplication of multiple observations on mother's natal year. We included the two-way interactions between mean SSTA in the natal maternal year and both the recruiting ages of daughters and sons and mean SSTA experienced by daughters and sons in early life (only the particular early years retained in the models described above; see “RESULTS”) and during each season when they bred. In these analyses we also included the two-way interactions between the mean SSTA in the natal maternal year and both the linear and quadratic terms for mother's age.

#### g) Differences between sexes

In order to assess whether long-term developmental effects of ENSO differ between females and males, we built additional models including both sexes to test for interactions between sex and SSTA whenever separate analyses of the two sexes revealed statistically significant effects in one or both of them. We also tested interactions between sex and SSTA in the natal maternal year in order to examine whether statistically significant transgenerational effects of ENSO differ between the sexes.

#### h) Model selection

We simplified models by sequentially dropping statistically non-significant interactions and main terms. To compare the simplified minimal adequate model with the model including a non-significant term or with the model excluding a significant term, we used χ^2^ tests for GLMMs and likelihood ratio tests for LMEs [72]. All models were fitted using R (Version 2.12.1 [73]). We report all significant terms and interactions (α≤0.05) retained in minimal adequate models (except random terms: cohorts, individuals, year of reproduction), along with mean ± standard error (SE) of estimated slopes (*β*).

## Results

### Recruiting age

**Figure 1:**
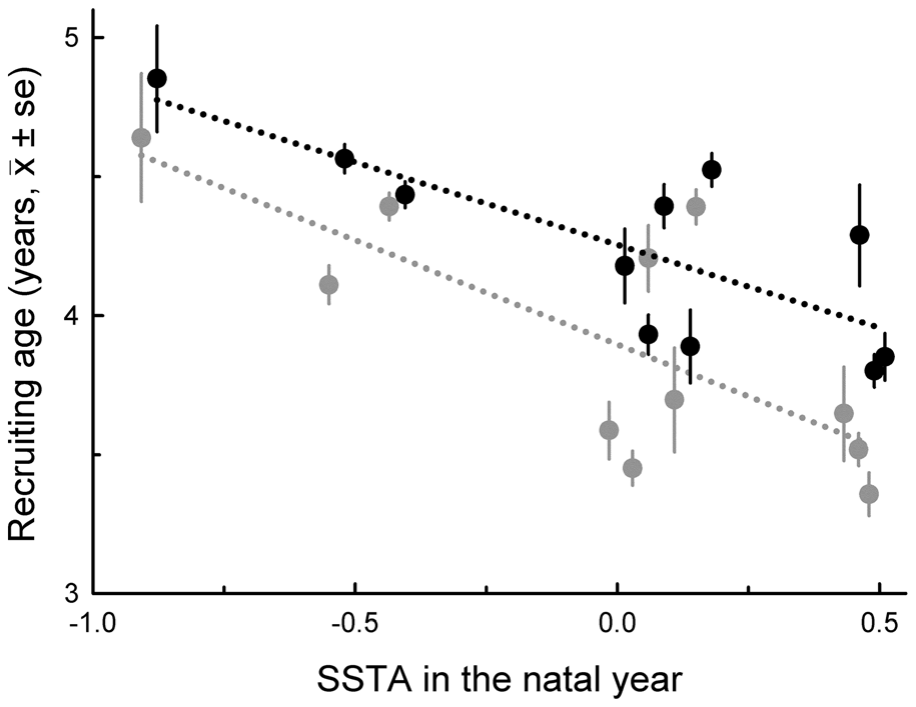
Mean recruiting age of 1189 female (shown in grey) and 1367 male (shown in black) boobies in relation to the annual mean sea surface temperature anomalies (SSTA) during their natal years (1988–2000). To simplify visualization, values of males are laterally displaced.

Contrary to expectation, the warmer the ocean (i.e., positive values of SSTA) during the year before birth, the natal year and the second year of life, the younger were females when they recruited into the population. Similarly, the warmer the ocean during the natal year and the second year of life, the younger were males when they recruited (Table 1, Figure 1).

**Table 1:** Minimal adequate GLMMs with cohort as a random effect examining the impacts of SSTA during the year before birth and each of the first three years of life on four life history parameters of 1189 female and 1367 male booby recruits up to age 10 years.

	Females			Males		
	Estimate (SE)	χ^2^	*P*	Estimate (SE)	χ^2^	*P*
**1. Recruiting age**						
Intercept	1.39 (0.02)			1.46 (0.02)		
SSTA in the year before birth	**–0.11 (0.03)**	**7.02**	**0.008**	–0.04 (0.03)	2.09	0.15
SSTA in the natal year	**–0.17 (0.03)**	**10.94**	**<0.001**	**–0.15 (0.03)**	**12.00**	**<0.001**
SSTA in the second year of life	**–0.13 (0.05)**	**4.89**	**0.030**	**–0.11 (0.04)**	**5.45**	**0.02**
SSTA in the third year of life	0.02 (0.05)	0.17	0.68	0.03 (0.04)	0.49	0.48
**2. Number of breeding attempts**						
Intercept	1.96 (0.08)			1.95 (0.07)		
SSTA in the year before birth	–0.03 (0.07)	0.15	0.70	0.04 (0.05)	0.44	0.51
SSTA in the natal year	**–0.18 (0.09)**	**3.64**	**0.056**	**–0.16 (0.07)**	**4.90**	**0.03**
SSTA in the second year of life	0.12 (0.09)	1.49	0.22	–0.08 (0.07)	1.14	0.29
SSTA in the third year of life	0.08 (0.11)	0.54	0.46	–0.06 (0.08)	0.56	0.45
**3. Total breeding success**						
Intercept	–1.03 (0.10)			–1.14 (0.10)		
SSTA in the year before birth	–0.11 (0.07)	1.92	0.16	–0.04 (0.07)	0.34	0.56
SSTA in the natal year	–0.004 (0.10)	0.00	1.00	–0.02 (0.09)	0.06	0.81
SSTA in the second year of life	–0.18 (0.11)	2.24	0.13	**–0.23 (0.09)**	**4.70**	**0.03**
SSTA in the third year of life	0.06 (0.11)	0.24	0.62	–0.03 (0.11)	0.10	0.75
**4. 10-year longevity**						
Intercept	0.99 (0.06)			0.99 (0.06)		
SSTA in the year before birth	0.02 (0.02)	1.18	0.28	0.009 (0.02)	0.20	0.65
SSTA in the natal year	0.03 (0.03)	1.02	0.31	**0.06 (0.02)**	**5.65**	**0.02**
SSTA in the second year of life	–0.02 (0.04)	0.36	0.55	0.01 (0.03)	0.12	0.73
SSTA in the third year of life	0.03 (0.03)	1.38	0.24	0.004 (0.03)	0.01	0.90

Full models are described in MATERIALS AND METHODS.

Values in bold represent the significant factors retained in minimal adequate models.

This advance in recruiting age does not seem to be due to cohorts born in El Niño years producing fewer recruits and consequently experiencing less competition to occupy breeding slots: the lower the number of recruits in a given cohort, the greater their average age at recruitment, although this relationship was not statistically significant (LM: *F* = 0.0015; *P* = 0.97, *N* = 11 cohorts).

The effect of ocean temperature during the year before birth on recruiting age differed with marginal significance between the sexes (SSTA during the year before birth x Sex: *β* = 0.08±0.04, χ^2^  = 3.65, *P* = 0.056), being limited to females. But the effects of temperature experienced in both the natal year and the second year of life did not differ significantly between the sexes (SSTA during the natal year x Sex: *β* = 0.02±0.05, χ^2^  = 0.17, *P* = 0.68; SSTA during the second year of life x Sex: *β* = 0.08±0.04, χ^2^  = 0.02, *P* = 0.90; *N* = 1189 females and 1367 males). Further, in the two sexes, SSTA had similar effects on recruiting age whether experienced in the year before birth, the natal year or the second year of life (comparison of slopes: *P* values: range  = 0.08–0.36).

### Number of breeding attempts up to age 10 years

Over the first 10 years, females and males, respectively, performed 1–8 and 1–11 breeding attempts, breeding an average 3.95±2.20 and 3.98±2.10 times (±SD). In both sexes, the warmer the ocean during the natal year, the fewer breeding attempts were made within 10 years, although in females this effect was only marginally significant (Table 1). On average, an extra 1.5°C in the natal year meant 1.15 and 1.27 fewer breeding attempts by females and males, respectively. In both sexes, this reduction in number of breeding attempts after higher natal temperatures did not appear to be the result of a shorter 10-year longevity but a diminished propensity to breed: although shorter 10-year longevity was associated with fewer breeding attempts (females: *β* = 0.14±0.005, χ^2^  = 756.84, *P*<0.001; males: *β* = 0.13±0.004, χ^2^  = 753.22, *P*<0.001), 10-year longevity was unaffected by ocean temperature during early life (see results for 10-year longevity below).

The effect of ocean temperature during the natal year on the number of breeding attempts did not differ between the sexes (SSTA during the natal year x Sex: *β* = 0.02±0.05, χ^2^  = 0.12, *P* = 0.73; *N* = 1189 females and 1367 males).

### Laying dates

**Figure 2:**
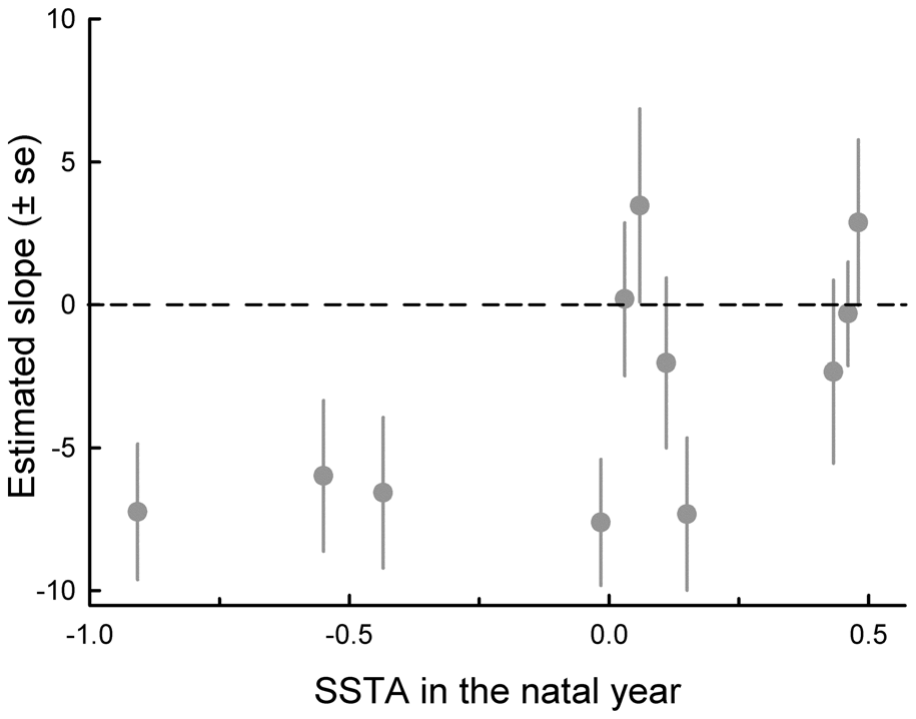
Slope of the regression of Julian laying date on age in relation to annual mean sea surface temperature anomalies (SSTA) during the natal years (1988–2000) of 1140 females.

Ocean temperature experienced in the prenatal year and the first three years of life had no effect on laying date in the season when males and females recruited (all *P* values >0.05). However, in females ocean temperature experienced in the natal year influenced the relationship between laying date and age (Age x SSTA during the natal year: *β* = 5.44±2.12, χ^2^  = 6.66, *P* = 0.01; *N* = 1140 females and 4272 attempts). Intriguingly, for females born in warm years there was no change in laying date with age, while females born in cold years advanced their laying date with age (Figure 2). This interaction between age and natal ocean temperature was confined to females (Age x SSTA during the natal year x Sex: *β* = –2.33±0.77, χ^2^  = 9.07, *P* = 0.003; *N* = 1140 females and 1328 males).

### Annual breeding success up to age 10 years

In both sexes, the earlier that breeders established their clutches, the higher their annual breeding success, both in the season when they recruited (Females: *β* = –1.48±0.11, χ^2^  = 158.45, *P*<0.001, *N* = 1007 females; Males: *β* = –1.62±0.10, χ^2^  = 267.27, *P*<0.001, *N* = 1155 males) and across all breeding attempts up to age 10 years (Females: *β* = –1.48±0.05, χ^2^  = 749.98, *P*<0.001, *N* = 1132 females and 4243 reproductions; Males: *β* = –1.45±0.05, χ^2^  = 883.15, *P*<0.001, *N* = 1315 males and 4955 reproductions).

We did not find any relationship between SSTA experienced by females and males in the prenatal year or any of the first three years of life and their standardized annual breeding success, either in the season when they recruited or across all breeding attempts up to age 10 years (Table 2).

**Table 2:** Minimal adequate LMEs with cohort and breeder identity (only in models with repeated measures) as random effects examining the impacts of SSTA during the year before birth and each of the first three years of life on the standardized annual breeding success of booby recruits in the season when they recruited^a^ and across all their breeding attempts up to age 10 years^b^.

	Females			Males		
Standardized annual breeding success	Estimate (SE)	χ^2^	*P*	Estimate (SE)	χ^2^	*P*
**a) In the recruiting season**						
Intercept	0.83 (0.07)			0.60 (0.14)		
SSTA in the year before birth	NS	2.05	0.15	NS	0.37	0.54
SSTA in the natal year	NS	0.1	0.75	NS	0.3	0.58
SSTA in the second year of life	NS	0.34	0.56	NS	1.99	0.16
SSTA in the third year of life	NS	0.2	0.65	NS	1.36	0.24
**b) Across all breeding attempts up to age 10 yrs**						
Intercept	0.21 (0.23)			0.44 (0.14)		
SSTA in the year before birth	NS	1.64	0.2	NS	0.43	0.51
SSTA in the natal year	NS	0.96	0.33	NS	0.1	0.75
SSTA in the second year of life	NS	0.26	0.61	NS	1.33	0.25
SSTA in the third year of life	NS	0.48	0.49	NS	2.53	0.11

a *N* = 1007 females and 1155 males.

b *N* = 1132 females and 1315 males.

Full models are described in Materials and Methods.

### Total breeding success up to age 10 years

Females and males, respectively, produced 0–12 and 0–14 fledglings in their first 10 years, averaging (± SD) 3.44±2.73 and 3.44±2.68 fledglings.

**Figure 3:**
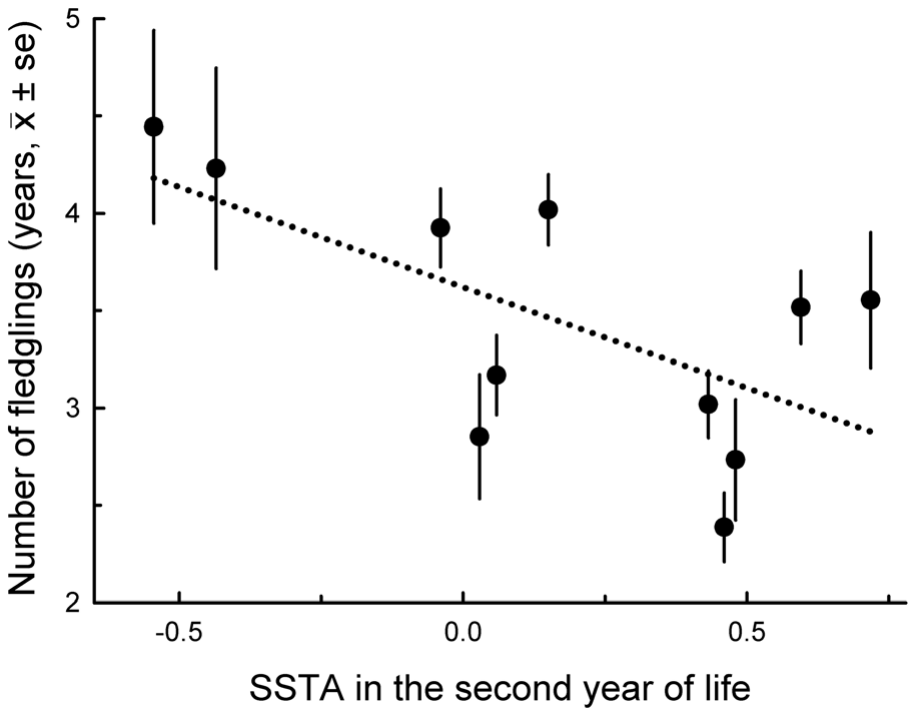
Mean accumulated reproductive success (i.e., number of fledglings) up to age 10 years of 1363 male boobies and the annual mean sea surface temperature anomalies during their second years of life.

In females, total 10-year (unstandardized) breeding success was unrelated to SSTA experienced in the prenatal year or any of the first three years of life; in males, total 10-year breeding success was unrelated to temperature in the prenatal, natal, and third years but declined with warmer ocean water during the second year of life (Table 1, Figure 3). This decline was confined to males (SSTA during the second year of life x Sex: *β* = –0.18±0.07, χ^2^  = 5.60, *P* = 0.02; *N* = 1181 females and 1363 males).

In both sexes, higher 10-year breeding success totals were associated with greater longevity in the 10-year period (Females: *β* = 0.16±0.01, χ^2^  = 109.88, *P*<0.001, *N* = 1181 females; Males: *β* = 0.17±0.01, χ^2^  = 139.58, *P*<0.001, *N* = 1363 males) and more breeding attempts (Females: *β* = 0.19±0.01, χ^2^  = 183.16, *P*<0.001; Males: *β* = 0.20±0.01, χ^2^  = 247.88, *P*<0.001).

The general absence of an effect of natal SSTA on total fledgling production despite a negative effect of SSTA on number of breeding attempts could be due to increasing stochastic variation in variables scored later in life. The slope of total fledgling production on natal SSTA estimated by multiplying the slope of the number of breeding attempts on SSTA by mean annual breeding success (Females: *β* = –0.17; Males: *β* = –0.15) was within the 95% confidence interval (CI) of the slope of total fledgling production on natal SSTA yielded by our model (Females: *β* = –0.004±0.10, [CI  = –0.192–0.184]; Males: *β* = –0.02±0.09, [CI  = –0.191–0.147]).

### 10-year longevity

On average (± SD), longevity of females and males over the 10 years was similar: 7.60±2.50 and 7.90±2.30 years, respectively. Only male longevity was related to ocean temperature in early life: contrary to our prediction, the warmer the ocean during a male's natal year, the longer he tended to live (Table 1). This effect was small and did not differ from the null effect of SSTA on 10-year longevity observed in females (SSTA during the natal year x Sex: *β* = 0.04±0.03, χ^2^  = 1.49, *P* = 0.22; *N* = 1181 females and 1366 males).

In both sexes, shorter longevity within 10 years was predicted by younger recruitment (Females: *β* = 0.11±0.01, χ^2^  = 91.67, *P*<0.001, *N* = 1181 females; Males: *β* = 0.12±0.01, χ^2^  = 107.43, *P*<0.001, *N* = 1366 males).

### Nestling growth of recruits

**Figure 4:**
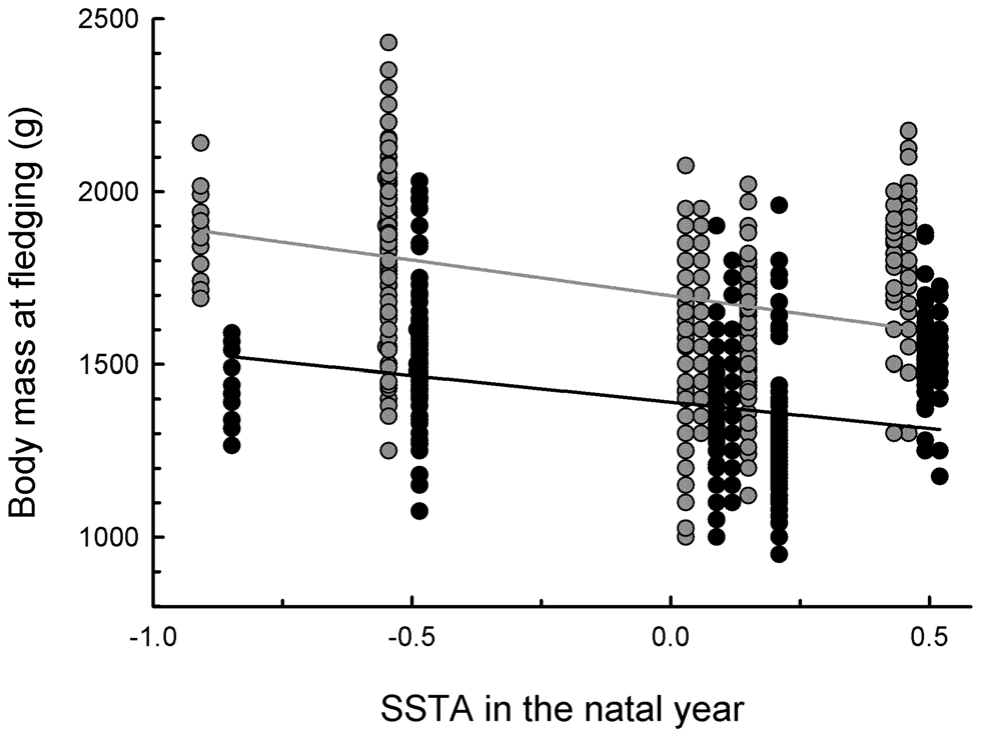
Body mass at age 70d of 670 female (shown in grey) and 746 male (shown in black) recruits in relation to the annual mean sea surface temperature anomalies (SSTA) during their natal years. To simplify visualization, values of males are laterally displaced.

Surprisingly, the warmer the ocean during the natal year, the longer were the ulnas of female and male recruits at fledging (age 70 d), although this effect was of negligible magnitude and only marginally significant (SSTA in the natal year: *F*_1, 2525_  = 3.68, *P* = 0.06). An increase in ocean temperature of 1.5°C in the natal year meant an increase of only 0.9 and 1.0 mm (i.e., an increase of 0.4 and 0.5%) in ulna length in females and males, respectively. The effect was similar for males and females (SSTA in the natal year x sex: *F*_1, 2525_  = 0.005, *P* = 0.94, *N* = 1174 females and 1351 males). By contrast, the warmer the ocean in the natal year, the lighter were the body masses of female and male recruits at fledging, a reduction that was greater in females, the larger sex, than in males (SSTA in the natal year x Sex: *β* = 53.76±25.47, *F*_1, 1416_  = 4.46, *P* = 0.035, *R* ^2^  = 0.47; *N* = 670 females and 746 males). An extra 1.5°C in the natal year meant a reduction in body masses of females and males of 207 g (16%) and 153 g (14%), respectively (Figure 4). However, body mass at fledging was not related to recruiting age, total number of breeding attempts, total breeding success or longevity up to age 10 years (all *P* values >0.05).

### Transgenerational effects

**Figure 5:**
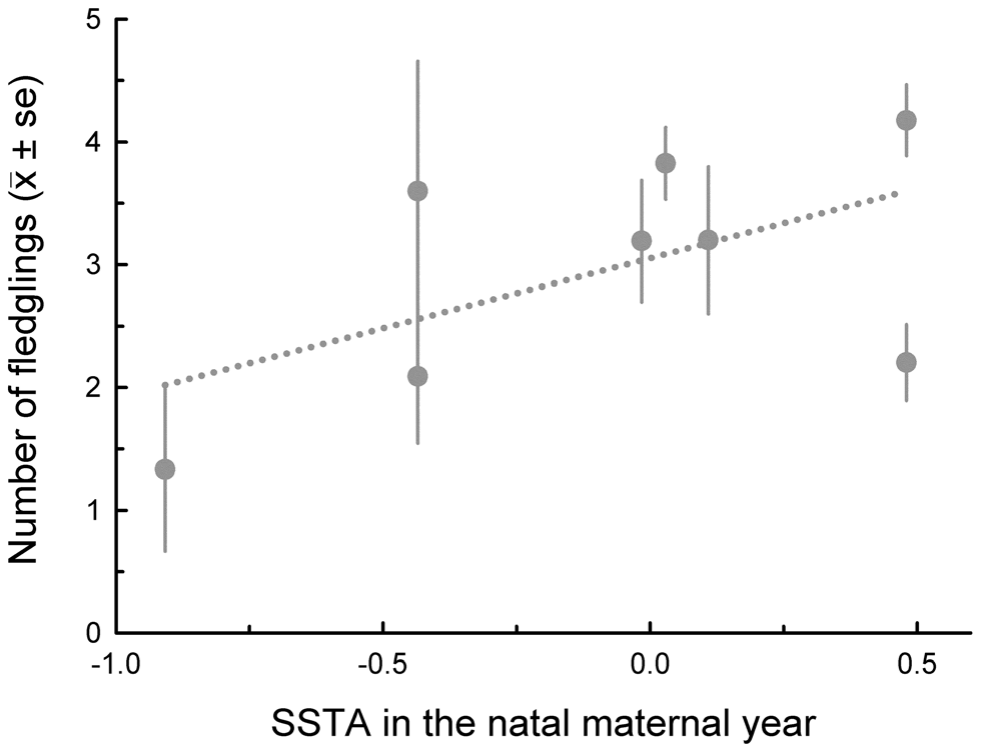
Mean accumulated reproductive success (number of fledglings) up to age 10 years of 330 female boobies in relation to the mean sea surface temperature anomaly during the natal years of their mothers.

Anomalies in ocean temperature experienced by mothers in their natal year did not affect their daughters' recruiting age, their 10-year breeding frequency or their laying dates (all *P* values >0.05), but were related to their breeding success. Although SSTA in the mother's natal year was not related to her daughters' standardized annual breeding success at recruitment (χ^2^  = 0.08, *P* = 0.77; *N* = 281 females), it was related to their standardized annual breeding success over the first ten years (*β* = 0.14±0.07, χ^2^  = 4.45, *P* = 0.03; *N* = 317 females and 1137 reproductions) and to their total 10-year breeding success (*β* = 0.16±0.08, χ^2^  = 3.89, *P* = 0.049; *N* = 330 females). Contrary to expectation, the warmer the ocean in a mother's natal year, the higher were both of those 10-year measures; on average, an extra 1.5°C in the mother's natal year meant an extra 1.5 total fledglings for her daughter (Figure 5). Nonetheless, the relationship between SSTA in a mother's natal year and her daughters' breeding success was not observed when we deleted 1988 from the analysis, the natal maternal year with lowest values of SSTA (Standardized annual breeding success: *β* = 0.13±0.08, χ^2^  = 2.77, *P* = 0.096; *N* = 296 females and 1063 reproductions; total 10-year breeding success: *β* = 0.17±0.10, χ^2^  = 2.87, *P* = 0.09; *N* = 308 females).

**Figure 6:**
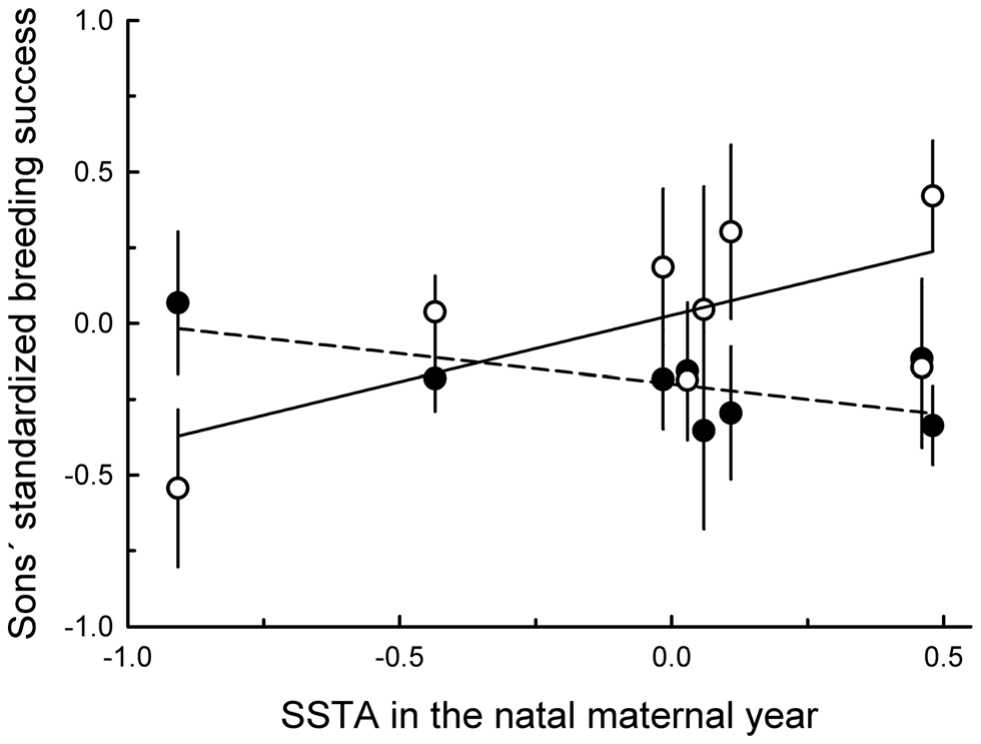
Standardized breeding success (mean ± se) in the first breeding attempt of 188 males recruiting at ages 3–4 years (closed dots, dashed line) and 133 males recruiting at ages 5–6 years (open dots, solid line) in relation to annual mean sea surface temperature anomalies during the natal years of their mothers. To simplify visualization, we show conditional plots from the estimated values of the interaction male recruiting age x SSTA in the mother's natal year.

Somewhat similarly for sons, oceanographic conditions experienced by mothers in their natal year were unrelated to recruiting age, 10-year breeding frequency or laying dates (all *P* values >0.05), but affected their sons' standardized annual breeding success at recruitment, depending on the age when the sons recruited (Recruiting age x SSTA in the natal maternal year: *β* = 0.35±0.11, χ^2^  = 9.14, *P* = 0.002; *N* = 321 males). The warmer the ocean during the mothers' natal year, the fewer fledglings produced at recruitment by sons that recruited younger (at ages 3–4 years) but the more fledglings produced at recruitment by sons that recruited older (at ages 5–6 years) (Figure 6). Nevertheless, this interaction between SSTA in the mother's natal year and recruiting age of her sons was not statistically significant when 1988, the natal maternal year with lowest values of SSTA, was deleted from the analysis (*β* = 0.24±0.14, χ^2^  = 2.97, *P* = 0.085; *N* = 299 males). In contrast with the finding for daughters, SSTA in the mother's natal year did not influence either her sons' standardized annual breeding success over the first ten years or their total 10-year (unstandardized) breeding success (all *P* values >0.05).

Two of the above three apparent differences in transgenerational effects of SSTA in the mother's natal year on daughters versus sons were confirmed by testing for interactions with sex. The effect on annual breeding success over the first ten years was limited to daughters, although the interaction was only marginally significant (SSTA during the natal maternal year x Sex: *β* = –0.17±0.10, χ^2^  = 3.08, *P* = 0.08; *N* = 317 females and 362 males). The interactive effect of SSTA and recruiting age on standardized breeding success at recruitment was specific to sons (Recruiting age x SSTA during the natal maternal year x Sex: *β* = 0.44±0.18, χ^2^  = 6.13, *P* = 0.01; *N* = 281 females and 321 males). However, testing failed to confirm that the positive effect of SSTA on total 10-year breeding success was confined to daughters (SSTA during the natal maternal year x Sex: *β* = –0.04±0.10, χ^2^  = 0.16, *P* = 0.69; *N* = 330 females and 375 males).

## Discussion

Our analyses suggest that ENSO-related conditions experienced by blue-footed boobies during the first two (pre-reproductive) years of life, or by their parents in the year before their birth, influence the timing of recruitment and several post-recruitment life-history traits, during at least the first 10 years after birth. Some of these effects differ between the sexes and effects on females cascade to the next generation. In analyses of annual breeding date and breeding success, none of the interactions between ocean temperature in any of the early years and temperature in the current breeding year was statistically significant, implying that effects of ENSO-related conditions in early life on reproduction are independent of effects of current ENSO events during particular breeding attempts. Similar independence of early developmental effects and current effects of environmental perturbations has been documented in other vertebrate species (e.g. [12], [29], [54] but see [6]).

Male and female boobies that experienced unfavorable warm water conditions in their natal and second years of life recruited earlier into the breeding population, as did females whose parents experienced warm water in the year before the females' birth, when their allocation of nutrients to upcoming reproduction could be constrained by current conditions. The opposite was expected since long-lived animals tend to postpone breeding until they are in sufficiently good condition to face costs of reproduction [74], and reduced body mass and size at fledging cause many birds to postpone recruitment [17], [75]. Evidently, boobies can bring forward their first attempt at breeding despite food limitation and other ENSO-related challenges constraining their parents' pre-breeding energetic balance, slowing nestlings' growth ([57], this study) or constraining juveniles' preparations for recruitment [56]. This implies that boobies respond to these early stresses with some combination of parental buffering, for example by adjusting provisioning [39], [76], compensatory growth [2] and physiological and metabolic adjustments during development [26], [77].

Rather than a strategic response, early recruitment of fledglings born in warm-water years could be a result of production of fewer recruits during El Niño years resulting in less competition to occupy breeding slots. However, the fewer recruits in a given cohort, the greater their average age at recruitment. Another alternative explanation is that earlier recruitment of fledglings from warm-water years is the result of annual variation in the quality of successful breeders and their offspring: in warm-water years the proportion of high quality breeders may increase if such adults are more likely to breed or more successful than other adults in such conditions [43]. Consistent with this idea, recruits from warm-water years were slightly larger (ulnas 0.4–0.5% longer) than recruits from cold-water years, but they were also 14–16% lighter, implying poor provisioning, so the evidence in this respect is equivocal.

Bringing the recruiting age forward in response to warm water in the natal year appears to represent strategic phenotypic adjustment by both males and females to maximize long-term fitness in the face of early stressful conditions [26], at least when results are assessed over the first 10 years of life. Although early recruitment of both sexes in this population predicts a shorter life (67), males and females that faced warm water in the natal year lived as long as those that faced cold water (slightly longer in the case of males) and in the first ten years achieved similar standardized annual breeding success and total breeding success despite breeding less frequently than them. By recruiting earlier and spreading ∼1.15–1.27 fewer breeding attempts over a similar period, fledglings from warm-water years managed to breed just as early in each season and eventually produce as many offspring in each attempt and in total as fledglings from cold-water years. The benefit of this strategy probably derives from blue-footed boobies' ability to initiate breeding in better condition when breeding attempts are more spaced out, specifically after skipping years [68].

Females showed evidence of additional complexity in their strategy: whereas those born in cold-water years advanced their laying date with age, those born in warm-water years maintained early laying throughout the first ten years, as if prepared for this by experiencing harsh conditions early in life. Thus, warm ENSO conditions experienced in the natal year apparently elicit phenotypic plasticity [78] that allows individuals to mitigate impacts on fitness [26], although trade-offs between traits may occur later in life [14]. Detection of potential trade-offs will require monitoring individuals beyond age 10 years and to the ends of their lives. Failure to find negative impacts of ENSO conditions on total fledgling production despite a reduction in the number of breeding attempts is puzzling and could be due to high stochastic variation in variables scored at later life-history stages and consequent reduced statistical power in analysing them. It is possible that there is a small reduction in total fledgling production that we failed to detect.

Warm water in the early years of life (except the third year) triggered an advancement of recruitment and there is evidence that this advancement was adaptive in both sexes although less so in males facing the warm water in their second year. Females brought forward their recruitment after experiencing warm water in the year before birth, the natal year or the second year of life; and they achieved similar annual and total reproductive success to females that experienced cold water, while maintaining similar frequencies and dates of breeding, and similar longevities. Males that experienced warm water in the natal year brought forward their recruitment, bred less frequently and tended to live longer; and they achieved similar annual and total reproductive success to males born in cold years. Males that faced warm water in the second year of life also advanced their recruitment but, despite living as long and breeding as frequently and early as males experiencing cold water in their second year, they underperformed in total reproductive success although not in standardized annual reproductive success. That number of breeding attempts, female laying dates and 10-year longevity of males were affected by warm water when experienced in the natal year but not when experienced in the following two years, supports Lindström's [1] suggestion that the earlier an individual's development is disturbed, the greater are the effects. On the other hand, recruiting age was similarly affected by warm water whether experienced in the year before birth, the natal year or the second year of life, and total breeding success of males was affected by warm water only when faced in the second year of life. The age of greatest susceptibility may depend on the variable of interest.

Recruits born in warm-water years were underweight at age 70 d, with females and males showing deficits of 16% and 14%, respectively, under an anomaly of +1.5°C. Adjustment of energy allocation during the late nestling period in warm-water years is implied by such underweight nestlings showing no deficit in skeletal size at age 70d. Similar adjustments in chick development in response to low food availability have recently been documented in the black noddy (*Anous minutes*) in the western tropical Pacific [48], suggesting that this could be a common adaptive response of long-lived seabirds to low or highly variable food availability in tropical oceans [48]. Surprisingly, low body mass at fledging did not prejudice timing of recruitment, longevity, breeding frequency or breeding success up to age 10 years, implying that the alternative life history adopted by fledglings from warm-water years was similarly effective for fledglings of all body masses.

We found some differences between males and females in the impacts of ENSO-related conditions on subsequent life-history traits, and these depended on the age when warm water was encountered. Warm water in the natal year induced both sexes to start breeding at an early age and breed relatively infrequently, as well as inducing a slight increase in longevity in males. In contrast, only in females did warm water in the natal year influence the relationship between laying date and age, possibly due to their greater reduction in body mass at fledging, itself attributable to greater body size and food needs of females [61], [79]. Warm water during the second year of life induced early breeding in both sexes but only males showed diminished total reproductive success over the first ten years. Males recruit (and may mature) nearly half a year older than females (Figure 1, [11]), so they may show more prolonged developmental sensitivity to perturbations than females. Other possible explanations are that females, being larger, mitigate the impact of second-year conditions by foraging farther and over larger areas [80]; or, being the egg-makers, fine-tune egg components [81] so as to favour offspring survival [25]. Finally, warm water in the year before birth induced early breeding only in females, suggesting that conditions experienced by parents during their preparation for breeding or by gravid mothers before laying may differentially influence the embryonic development of female offspring [25], [82]. Candidate mechanisms include sex differences in temperature requirements during incubation [83] or in concentrations of nutrients and hormones in the egg [84].

We also found evidence for sex-specific maternal transgenerational effects of ENSO. During the first ten years of life, daughters of females that experienced warm ENSO conditions in their natal year showed improved annual and total breeding success. By contrast, sons of females that experienced ENSO conditions in their natal year showed reduced breeding success in their first attempt if they recruited young and an increase in this same trait if they recruited after age 5 years. These effects may reflect maternal (and grandmaternal) buffering of environmental impacts on developing offspring [25], possibly mediated by epigenetic mechanisms [31]. Benefits of this proposed buffering were greater in females, the faster-growing sex that is more vulnerable to energetic stress in infancy and larger-bodied in adulthood. However, it is not clear to us why daughters should benefit from transgenerational buffering more than sons do. Sex-specific transgenerational effects could be due to facultative adjustments of relative maternal expenditure on daughters and sons [13], [85], differential sensitivity of the sexes to environmental stresses during their embryonic development [82], or mediation of transgenerational effects by sex chromosomes [86]. We note that the correlations providing evidence for transgenerational effects of ENSO were loose and their significance depended on the inclusion of a year (1988) with very low SSTA, casting some doubt on these effects. The causes of transgenerational effects of ENSO on the two sexes and their consequences on life-history evolution and population dynamics deserve detailed analysis in further studies.

Our findings contribute to the scarce evidence of long-term fitness consequences of natal conditions in wild populations. They demonstrate for the first time that animals can respond to ENSO-related environmental challenges during their early development by plastically adopting alternative life-history strategies, and suggest that these alternative strategies may be equally successful in some circumstances. They also show that plastic variation in life history strategies of the blue-footed booby hinges on the developmental stage when perturbation is faced [2] and the sex of affected individuals [10], [13], and that the net fitness effects of alternative strategies probably depend on the extent of covariation among life-history traits [6].
